# LightEdu-Net: Noise-Resilient Multimodal Edge Intelligence for Student-State Monitoring in Resource-Limited Environments

**DOI:** 10.3390/s25247529

**Published:** 2025-12-11

**Authors:** Chenjia Huang, Yanli Chen, Bocheng Zhou, Xiuqi Cai, Ziying Zhai, Jiarui Zhang, Yan Zhan

**Affiliations:** 1 National School of Development, Peking University, Beijing 100871, China; 2 China Agricultural University, Beijing 100083, China; 3 Artificial Intelligence Research Institute, Tsinghua University, Beijing 100084, China

**Keywords:** LightEdu-Net, state recognition, multimodal sensor fusion, edge-based educational perception, educational economics

## Abstract

Multimodal perception for student-state monitoring is difficult to deploy in rural classrooms because sensors are noisy and computing resources are highly constrained. This work targets these challenges by enabling noise-resilient, multimodal, real-time student-state recognition on low-cost edge devices. We propose LightEdu-Net, a sensor-noise-adaptive Transformer-based multimodal network that integrates visual, physiological, and environmental signals in a unified lightweight architecture. The model incorporates three key components: a sensor noise adaptive module (SNAM) to suppress degraded sensor inputs, a cross-modal attention fusion module (CMAF) to capture complementary temporal dependencies across modalities, and an edge-aware knowledge distillation module (EAKD) to transfer knowledge from high-capacity teachers to an embedded-friendly student network. We construct a multimodal behavioral dataset from several rural schools and formulate student-state recognition as a multimodal classification task with explicit evaluation of noise robustness and edge deployability. Experiments show that LightEdu-Net achieves 92.4% accuracy with an F1-score of 91.4%, outperforming representative lightweight CNN and Transformer baselines. Under a noise level of 0.3, accuracy drops by only 1.1%, indicating strong robustness to sensor degradation. Deployment experiments further show that the model operates in real time on Jetson Nano with a latency of 42.8 ms (23.4 FPS) and maintains stable high accuracy on Raspberry Pi 4B and Intel NUC platforms. Beyond technical performance, the proposed system provides a low-cost and quantifiable mechanism for capturing fine-grained learning process indicators, offering new data support for educational economics studies on instructional efficiency and resource allocation in underdeveloped regions.

## 1. Introduction

Against the backdrop of rapidly advancing digital education, classroom instruction is moving from experience-driven practices toward data-driven and intelligent decision-making paradigms [1]. Real-time monitoring of student attention, fatigue, and classroom engagement can help teachers track learning dynamics, adjust instructional strategies, and provide an empirical basis for personalized support and resource allocation [2]. However, in rural and under-resourced areas, persistent issues such as insufficient teaching staff, limited classroom supervision, and delayed instructional feedback make it difficult to obtain such fine-grained behavioral information [3]. Although multimodal sensing and artificial intelligence techniques now enable automated analysis of student states from visual, physiological, and environmental data [4], most existing systems assume reliable sensors, stable networks, and cloud- or GPU-based computation, which rarely hold in these settings. Low-cost devices in real classrooms must cope with severe sensor noise, heterogeneous sampling rates, and strict constraints on computation, memory, and energy. As a result, intelligent tutoring systems, while theoretically valuable for large classes and high teaching workloads [5], remain hard to deploy in the very schools that would benefit most from them. This gap between methodological advances and deployable solutions motivates the design of LightEdu-Net, a multimodal perception framework explicitly tailored to noisy sensors and resource-constrained edge hardware in real rural classrooms.

Although intelligent tutoring systems have achieved notable methodological progress, many existing approaches remain difficult to apply in real classrooms, especially in rural schools [6]. Early work on student behavior monitoring typically relied on single-modal signals (e.g., camera images or wearable physiological data) and traditional machine learning models such as SVM, random forest, and KNN operating on handcrafted features [7,8]. These shallow representations struggle to capture the complexity and variability of classroom environments and are highly sensitive to illumination changes, occlusions, and sensor noise, leading to poor robustness in deployment [9,10]. Moreover, low-dimensional features are inadequate for describing temporal dynamics such as attention fluctuation and cumulative fatigue [11], and most pipelines assume desktop or server-side processing, which prevents real-time inference on the low-cost, low-power devices typically available in rural settings [12].

With the advancement of deep learning, student behaviors have increasingly been modeled using convolutional neural networks, Transformer architectures, and graph neural networks [13]. CNNs and Transformers can extract rich spatial–temporal features from visual, speech, and physiological signals, and multimodal fusion strategies such as early fusion, late fusion, and cross-attention improve recognition accuracy by exploiting complementary information across modalities [14,15]. However, most of these models are computationally heavy, rely on high-performance GPUs or cloud infrastructure, and assume relatively clean and homogeneous multimodal data [16,17]. In contrast, sensors in rural classrooms often produce noisy, low-fidelity, and asynchronously sampled signals, and cloud-based solutions may be unreliable due to limited bandwidth and raise privacy concerns [18]. Recent edge-oriented multimodal frameworks such as TinyM^2^Net, Farm-LightSeek, EduVQA, and lightweight maritime recognition systems demonstrate that compact models can be deployed on resource-constrained hardware in other domains [19,20], but there is still a lack of designs that jointly address sensor noise, heterogeneous educational modalities, and strict edge-computing constraints in real classrooms. This gap motivates our development of LightEdu-Net.

In this study, we focus on how to perform reliable multimodal student-state recognition in rural classrooms where sensing is noisy and computation must run on low-power edge devices. To address this problem, we propose LightEdu-Net, a lightweight multimodal framework designed to be both noise-resilient and deployable. The framework integrates hybrid feature encoding, cross-modal attention, and edge-aware optimization, enabling high-accuracy and low-latency inference on embedded platforms. The main contributions of this work are threefold.

1. We construct a multimodal behavioral dataset from rural schools, capturing visual, physiological, and environmental signals under real classroom conditions. The dataset explicitly contains noise artifacts, illumination changes, and motion disturbances, providing realistic supervision for evaluating robustness in resource-constrained environments.
2. We design LightEdu-Net, a sensor-noise adaptive multimodal network that combines a lightweight convolution–Transformer backbone with a sensor noise adaptive module (SNAM) and a cross-modal attention fusion module (CMAF). This architecture suppresses abnormal sensor inputs, aligns heterogeneous modalities, and extracts discriminative temporal–spatial features for student-state recognition.
3. We validate the framework through targeted experiments that reflect the core challenge: robustness and deployability. Results show that LightEdu-Net maintains high accuracy under increasing noise levels and operates in real time on edge devices such as Jetson Nano, Raspberry Pi 4B, and Intel NUC, demonstrating its suitability for practical deployment in resource-constrained educational settings.
4. From an educational economics perspective, the proposed framework provides low-cost and quantifiable learning process indicators for resource-constrained classrooms, enabling data-driven analysis of teaching efficiency and educational resource utilization in underdeveloped regions.

The remainder of this manuscript is organized as follows. Section 2 reviews related work on intelligent education, multimodal sensor fusion, edge computing, and the educational economics perspective that motivates our focus on rural and resource-constrained classrooms. Section 3 describes the materials and methods, including the construction of the multimodal educational behavior dataset, data preprocessing and augmentation strategies, and the proposed LightEdu-Net framework with its sensor noise adaptive module, cross-modal attention fusion module, and edge-aware knowledge distillation module. Section 4 presents the experimental setup and results, comparing LightEdu-Net with representative baselines, reporting ablation studies and noise-robustness analyses, and evaluating deployment performance on different edge devices, followed by a discussion of practical implications and remaining limitations. Section 5 concludes the paper and outlines directions for future research.

## 2. Related Work

### 2.1. Applications of Deep Learning in Intelligent Education

To understand why reliable multimodal perception remains challenging in real classrooms, we first review how deep learning has been applied in intelligent education. The application of deep learning in this field primarily relies on convolutional neural networks, Transformer architectures, and graph neural networks, each modeling student behavior from spatial, temporal, or relational perspectives [21,22]. Convolutional neural networks extract posture variations, facial expression details, and behavioral patterns from video frames through local receptive fields and hierarchical feature mechanisms, offering stable performance in attention monitoring, classroom behavior recognition, and emotion analysis [23]. With the emergence of attention mechanisms, Transformer models leverage global dependency modeling to capture dynamic behavioral changes over time and are particularly effective for states with strong temporal characteristics, such as attention fluctuation and cumulative fatigue [24]. Graph neural networks construct student–student or student–environment relational structures, enabling modeling of pose keypoint relationships and group learning behaviors [25].

Although these deep architectures significantly improve accuracy in educational behavior analysis, their limitations remain notable for deployment in resource-constrained schools. First, their large parameter sizes and high computational complexity generally require GPU acceleration to achieve real-time inference [26]. Second, many of these models are trained on controlled or high-quality datasets and therefore struggle to generalize to rural classrooms characterized by illumination variation, occlusions, missing data, and noise [27]. Third, incorporating multimodal signals further increases architectural complexity, making it difficult for such models to execute smoothly on embedded devices [28]. These constraints hinder the large-scale adoption of deep learning technologies in the very environments that most need support and motivate the lightweight, noise-resilient multimodal modeling focus of this study.

### 2.2. Multimodal Sensor Fusion Techniques

Reliable student-state recognition in real classrooms often requires combining multiple noisy and heterogeneous signals, making multimodal sensor fusion a central topic in intelligent education research. The primary objective is to integrate visual, physiological, speech, and environmental signals to obtain more comprehensive and stable representations than those provided by any single modality [29]. Early fusion techniques concatenate raw signals or low-level features at the input stage to exploit cross-modal complementarity [30], whereas late fusion aggregates decisions from independently encoded modalities and offers structural simplicity and interpretability [31]. More recently, cross-modal attention mechanisms have been employed to compute mutual information among modalities and adaptively adjust fusion weights, enabling the capture of complex inter-modal relationships in dynamic classroom environments [32]. These methods generally aim to enhance recognition accuracy and robustness [33].

However, when moving from controlled lab settings to rural classrooms, modality heterogeneity, inconsistent sampling frequencies, missing data, and diverse noise sources significantly increase the difficulty of fusion [34]. Simple concatenation or fixed fusion rules are often brittle under such conditions, and even attention-based approaches may waste capacity on corrupted channels if noise is not explicitly modeled. Effective multimodal fusion therefore requires not only expressive feature representations but also mechanisms that remain stable under varying data quality. This motivates the cross-modal attention fusion in LightEdu-Net, which is coupled with sensor-noise adaptation to dynamically adjust fusion strategies when confronted with low-quality, noisy, or heterogeneous classroom data.

### 2.3. Edge Computing and Lightweight Model Research

With the growing adoption of edge computing, many studies have sought to deploy deep learning models on embedded or low-power devices to reduce cloud dependence, improve real-time performance, and enhance data privacy protection [35]. The core idea of edge computing is to relocate part or all of the inference workload to devices close to the data source, thereby reducing communication overhead and increasing system autonomy [36]. However, the computational and storage requirements of most deep models substantially exceed the capacity of typical edge hardware, prompting the development of model compression and lightweight design techniques [37]. Pruning removes redundant weights to decrease computation, quantization reduces numerical precision to save memory, knowledge distillation transfers information from a large teacher model to a compact student model, and lightweight architectures (e.g., MobileNet, ShuffleNet) optimize structural efficiency by design [38]. These techniques have demonstrated strong performance in image classification, speech recognition, and various edge AI applications such as medical monitoring and traffic management [39].

Nevertheless, multimodal perception tasks are inherently more complex than unimodal tasks, and input heterogeneity often causes compressed models to struggle in maintaining both accuracy and real-time performance on constrained devices. Moreover, most existing lightweight approaches do not explicitly account for the noisy, distribution-unstable, and modality-inconsistent characteristics of educational environments, leading to potential instability when deployed in real classrooms. This gap motivates the edge-aware design in LightEdu-Net, where knowledge distillation and architecture choices are tailored to multimodal, noise-prone student-state recognition on low-power hardware.

### 2.4. Educational Economics Perspectives on Intelligent Tutoring Systems

Multimodal intelligent tutoring systems demonstrate substantial potential from an educational economics perspective, particularly in improving instructional quality and promoting educational equity in resource-constrained settings [40]. Rural schools commonly face systemic challenges such as insufficient teaching staff, oversized classes, and delayed instructional feedback, which reduce the effectiveness of existing human and material inputs [41]. Lightweight models that can be deployed on edge devices without reliance on high-bandwidth networks or high-performance computing facilities provide timely feedback for teachers and effectively enhance the utilization efficiency of scarce classroom resources [42]. From a cost–effectiveness standpoint, such technologies require relatively modest hardware investments compared to server- or cloud-based solutions, while the associated performance gains can exceed those of many traditional pedagogical interventions, making them particularly attractive for low-resource environments [43]. Consequently, intelligent tutoring systems are not only technological innovations within computer science but also serve as new measurement instruments and intervention tools for educational economics, facilitating more equitable educational opportunities and supporting evidence-based optimization of educational resource allocation across regions [44]. In this context, a noise-resilient, edge-deployable framework such as LightEdu-Net is especially relevant, as it is explicitly designed to operate under the economic and infrastructural constraints that characterize under-resourced schools.

## 3. Materials and Method

### 3.1. Data Collection

**Figure 1:**
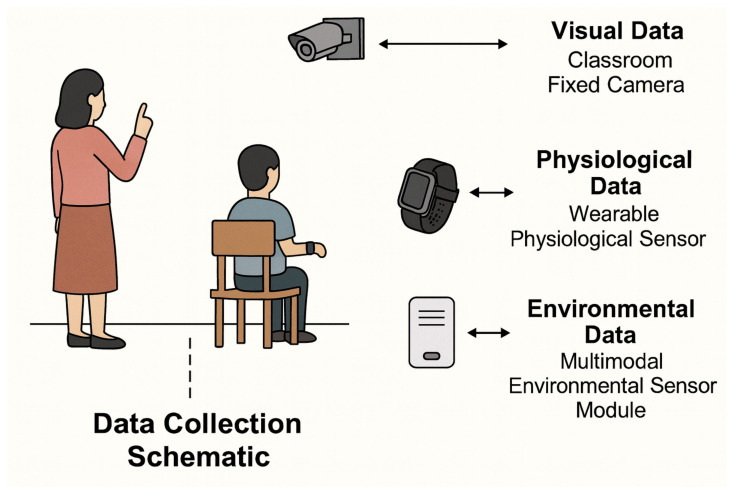
Overview of the multimodal data collection framework used in this study.

The data collection process was conducted from March 2024 to January 2025 across authentic classroom environments in three rural primary schools and one county-level middle school in western China, as shown in Table 1 and Figure 1. A total of 12 multimodal acquisition terminals were deployed, integrating high-definition cameras, wearable physiological sensors, and environmental monitoring units. This setting reflects the conditions under which LightEdu-Net is expected to operate: highly heterogeneous signals, frequent illumination changes, and unstable network connectivity. Visual data were captured using 1080p cameras mounted at the front and sides of classrooms at 30 FPS, providing continuous observation of posture, facial expressions, and gaze direction. Automatic exposure control and illumination compensation were enabled to cope with alternating natural and artificial light. Each 40-min lesson yielded approximately 72,000 sequential frames per camera, and infrared auxiliary lighting was used to improve face and keypoint visibility under dim or reflective conditions.

In total, data were collected from 268 students across Grades 3–9, with class sizes ranging from 32 to 48 students. The cohort includes both boys and girls and reflects the typical demographic composition of rural schools in western China in terms of age distribution and socio-economic background. Classroom subjects covered Chinese language, mathematics, English, and science, as well as a smaller number of sessions in moral education and integrated practical activities, thereby capturing a variety of instructional formats (lecture, discussion, group work, and individual practice).

Physiological signals were recorded through multi-channel wearable devices attached to students’ wrists or arms, providing heart rate (HR), electrodermal activity (EDA), temperature (TEMP), and blood oxygen saturation (SpO_2_) at 10 Hz. Data were streamed via Bluetooth 5.0 to a local edge node and synchronized with visual data. A caching and resume mechanism was used to address intermittent intranet connectivity, ensuring completeness. To reduce inter-individual variability, physiological signals were normalized and smoothed before model input. Environmental conditions were monitored at 1-second intervals using sensor modules placed at the front, rear, and window areas of the classroom. Measured variables included illumination (lux), noise level (dB), temperature (°C), and humidity (RH). These signals capture rapid fluctuations associated with instructional activities, such as reading aloud, classroom transitions, or external disturbances.

All data collection procedures were approved by the institutional ethics committee of the collaborating university and the local education authorities. Written informed consent was obtained from school administrators, teachers, and the legal guardians of all participating students, and assent was obtained from the students themselves. During acquisition and subsequent processing, personal identifiers were removed or anonymized, and only aggregated indicators are used in analysis and system feedback to protect privacy.

### 3.2. Dataset Processing

Reliable multimodal modeling requires that input signals exhibit comparable scale, temporal consistency, and noise stability. Classroom environments inherently contain illumination fluctuation, partial occlusions, loose-wearing artifacts, and motion-induced perturbations. To ensure that the downstream lightweight architecture receives stable inputs, we apply a hybrid processing strategy that combines basic signal filtering and normalization with targeted augmentation. These operations are not intended as contribution points, but rather provide necessary signal conditioning for the subsequent SNAM and CMAF modules to operate effectively under resource constraints.

#### 3.2.1. Time-Series Data

For physiological signals, filtering represents the first step to suppress power-line interference and high-frequency noise. Using linear time-invariant filtering expressed in convolutional form, the filtered signal is given by

(1) $$y\left(t\right)=\int_{-\infty }^{+\infty }x\left(\tau \right)h\left(t-\tau \right)\;d\tau ,$$

where x(t) is the raw signal and h(t) the impulse response of the filter. In practice, band-pass filters retain cognitive-related frequencies (e.g., 0.1–40 Hz) while removing out-of-band disturbances commonly observed in wearable classroom measurements.

Since inconsistent magnitudes across modalities may disturb optimization, all physiological and visual features are standardized. The standardized signal is defined as

(2) $$\hat{x}=\frac{x-\mu }{\sigma },$$

where μ and σ denote mean and standard deviation. To accommodate non-stationary statistics in long classroom recordings, a sliding-window standardization strategy is adopted:

(3) $${\hat{x}}_{t}=\frac{x_{t}-\mu_{t}^{\left(W\right)}}{\sigma_{t}^{\left(W\right)}},$$

where $\mu_{t}^{\left(W\right)}$ and $\sigma_{t}^{\left(W\right)}$ are the window-based mean and variance computed within *W* samples.

Sudden spikes caused by electrode displacement or motion artifacts are further smoothed by windowed averaging,

(4) $${\tilde{x}}_{t}=\frac{1}{\left|W\right|}\sum_{i=t-k}^{t+k}x_{i},$$

where |W|=2k+1. This preserves global trends while attenuating high-frequency jitter, producing temporally coherent features that SNAM later refines via learnable denoising.

#### 3.2.2. Image Data

Visual signals are augmented to simulate realistic disturbances. Brightness perturbation is modeled by a linear intensity transformation:

(5) $$I^{\prime }=\gamma I+\delta ,$$

where γ scales global brightness and δ models ambient light shift. This augmentation improves robustness to lighting variance often observed in classrooms with windows or ceiling lights.

Occlusion is simulated using a Cutout strategy. A random rectangular region Ω is selected and replaced:

(6) $$I^{\prime }\left(x,y\right)=\left\{\begin{array}{cc} 0, & (x,y)\in \Omega ,\\ I(x,y), & \mathrm{otherwise}. \end{array}\right.$$

This encourages the backbone to rely on multiple cues rather than single facial or postural regions, improving resilience when students move, lean, or are partially blocked.

### 3.3. Data Enhancement

To reduce over-sensitivity to sampling jitter, a temporal perturbation strategy is introduced. For each time step, a small offset $\epsilon_{t}$ is added:

(7) $$x_{t}^{\prime }=x_{t+\epsilon_{t}},$$

where $\epsilon_{t}$ follows a zero-mean, small-variance distribution. Such temporal jittering prevents overfitting to rigid time alignment and simulates realistic sensor drift.

Multimodal signals often differ in sampling rate. To allow cross-modal attention to operate on a unified temporal axis, all modalities are resampled via interpolation. For an original signal x(t) and resampled timeline $t^{\prime }$, we compute

(8) $$x^{\prime }\left(t^{\prime }\right)=\mathrm{Interp}\left(x\left(t\right),t^{\prime }\right),$$

where Interp(·) denotes linear interpolation. This temporal synchronization ensures that CMAF processes inputs within consistent windows, and that modality interactions reflect true behavioral dynamics rather than sampling discrepancies.

Taken together, these preprocessing steps provide stable and comparable multimodal inputs. They do not constitute novel algorithmic contributions, but they are essential to enable SNAM to learn frequency-domain denoising and CMAF to exploit cross-modal mutual information under noisy classroom conditions and edge-computing resource limits.

### 3.4. Proposed Method

#### 3.4.1. Overall

We propose LightEdu-Net, a noise-resilient multimodal student-state recognition framework designed for edge deployment in real classrooms. The model takes temporally aligned visual, physiological, and environmental features as input and passes them through three lightweight modality-specific encoders: a convolution–Transformer hybrid backbone for visual signals, a 1D convolutional–recurrent backbone for physiological signals, and multi-channel convolutions for environmental measurements. The encoded features are then refined by the sensor noise adaptive module (SNAM), which performs learnable band-pass denoising and channel reweighting to improve the signal-to-noise ratio before fusion. The denoised modality features are subsequently fed into the cross-modal attention fusion (CMAF) layer, where intra-modal attention emphasizes salient temporal segments and inter-modal attention captures complementary dependencies across modalities, yielding a compact joint representation. On top of this fused representation, a lightweight prediction head produces student-state labels suitable for classroom monitoring on embedded devices. During training, an edge-aware knowledge distillation (EAKD) scheme is applied, in which a high-capacity teacher network guides the LightEdu-Net student to approximate its decision boundaries while keeping the student architecture small and quantization-friendly. At inference time, only the student network is used: data flow sequentially through the encoders, SNAM, CMAF, and the prediction head, enabling robust multimodal perception with low latency on low-power edge hardware.

#### 3.4.2. Sensor Noise Adaptive Module

**Figure 2:**
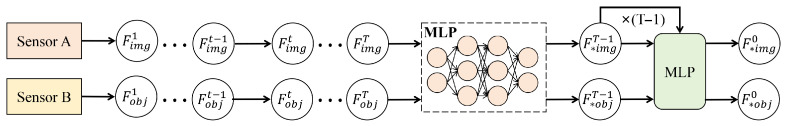
This figure illustrates the overall workflow of the sensor noise adaptive module (SNAM).

The sensor noise adaptive module (SNAM) is inserted between the modality-specific encoders and the multimodal fusion layer, following the noise-rectification pipeline illustrated in Figure 2. For each modality, the encoder output is first projected to a compact temporal feature sequence. Concretely, the visual backbone produces a tensor $V\in R^{T\times 14\times 14\times 64}$, which is passed through a 1×1 pointwise convolution (32 output channels) and global spatial average pooling to obtain $\tilde{V}\in R^{T\times 32}$. The physiological branch yields $\tilde{P}\in R^{T\times 64}$ through one-dimensional convolutions and GRU layers, and the environmental branch produces $\tilde{E}\in R^{T\times 32}$ via multi-channel convolutions. SNAM processes each modality feature sequence $X\in \{\tilde{V},\tilde{P},\tilde{E}\}$ independently with shared parameters, so that the same denoising mechanism is applied to visual, physiological, and environmental signals.

For a given modality, we first apply a lightweight temporal encoder to capture local dynamics and smooth high-frequency fluctuations. A depthwise–separable temporal convolution stack DS − TCN is used,

(9) Z=DS − TCN(X),

where two depthwise–separable Conv1d layers (kernel sizes k=5 and k=3, dilations d=1 and d=2) with LayerNorm and SiLU activation preserve the shape $Z\in R^{T\times C}$ (with C∈{32,64}). To further suppress noise in the spectral domain, we perform a learnable band-pass filtering. Let $F_{t}$ denote the FFT along the temporal axis and $\hat{Z}=F_{t}\left(Z\right)$ the corresponding spectrum. We define a parameterized mask

(10) $$M\left(\omega \right)=\sigma \left(s(\omega -\omega_{\mathrm{low}})\right)\;\sigma \left(s(\omega_{\mathrm{high}}-\omega )\right),$$

where $\omega_{\mathrm{low}}$ and $\omega_{\mathrm{high}}$ are learnable cutoff frequencies, s>0 is a learnable slope, and σ(·) denotes the sigmoid function. The filtered features in the time domain are then obtained by

(11) $$U=F_{t}^{-1}\left(M⊙\hat{Z}\right),$$

which attenuates frequency components outside the band of interest while preserving the temporal resolution.

Residual noise is often concentrated in a subset of channels, especially under sensor jitter or loose wearing. To adaptively down-weight such channels, we introduce a channel reweighting unit. Global temporal average pooling ${\mathrm{GAP}}_{t}$ followed by two bottleneck fully connected layers produces a set of channel importance scores $w\in R^{C}$:

(12) $$w=\sigma \left(W_{2}\;\phi \left(W_{1}\;{\mathrm{GAP}}_{t}\left(U\right)\right)\right),\;\;\;\;\;W_{1}\in R^{\frac{C}{r}\times C},\;W_{2}\in R^{C\times \frac{C}{r}},$$

where ϕ(·) is the SiLU activation and r=8 is the reduction ratio. The final SNAM output is

(13) Y=U⊙w,

which preserves the temporal dimension *T* and channel dimension *C* of the input. Channels dominated by noise receive smaller weights, whereas informative channels are retained.

To stabilize training and encourage meaningful masks, we introduce two auxiliary objectives. A simple spectral regularization penalizes excessive suppression of useful bands,

(14) $$L_{\mathrm{band}}=∥\left(1-M\right)⊙\hat{Z}{∥}_{2}^{2},$$

where *M* is the learnable frequency mask and $\hat{Z}$ is the temporal spectrum of the intermediate features. In addition, when teacher features or clean augmented views are available, we encourage the denoised features to stay close to a reference representation. Let $Y\in R^{T\times C}$ denote the SNAM output for the current modality, where $Y_{t,c}$ is the value at time step *t* and channel *c*, and let $T\in R^{T\times C}$ denote the corresponding teacher features (or clean augmented views) extracted at the same layer and temporal resolution, with $T_{t,c}$ aligned to the same position. The alignment loss is then defined as

(15) $$L_{\mathrm{align}}=\frac{1}{TC}\sum_{t,c}|Y_{t,c}-T_{t,c}|.$$

In the overall LightEdu-Net framework, the modality-wise outputs $Y^{\left(v\right)}\in R^{T\times 32}$, $Y^{\left(p\right)}\in R^{T\times 64}$, and $Y^{\left(e\right)}\in R^{T\times 32}$ are concatenated and projected to a unified embedding dimension before being fed into the cross-modal attention fusion module. In this rectification-before-fusion design, SNAM improves the signal-to-noise characteristics of each modality so that CMAF can allocate its attention capacity to physically meaningful temporal patterns rather than corrupted sensor artifacts, which is particularly important in noisy, resource-constrained classroom environments.

#### 3.4.3. Cross-Modal Attention Fusion

The cross-modal attention fusion (CMAF) module is designed to model interactive representations across modalities rather than within a single modality. Conventional self-attention computes dependencies using query, key, and value matrices (Q,K,V) derived from the same input, and thus primarily captures intra-modal structure. In contrast, CMAF explicitly exchanges information among visual, physiological, and environmental branches, allowing each modality to attend to others in a shared embedding space.

**Figure 3:**
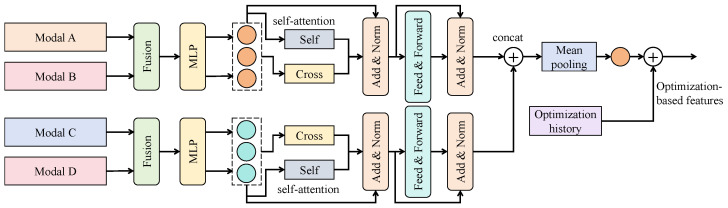
Architecture of the cross-modal attention fusion (CMAF) module.

As illustrated in Figure 3, CMAF operates on the SNAM-processed features and consists of stacked Transformer-style blocks. Each block contains intra-modal self-attention, inter-modal cross-attention, residual connections with LayerNorm, and a two-layer multilayer perceptron (MLP). The visual, physiological, and environmental branches receive inputs of shape $T\times C_{m}$, where *T* denotes the sequence length and $C_{m}\in \left\{32,64,32\right\}$ is the channel dimension for the three modalities, respectively. All modality-specific features are first projected to a unified embedding dimension d=96 so that they can be processed jointly. In our implementation, CMAF uses three such blocks with h=8 attention heads per block and head dimension $d_{h}=d/h=12$.

Formally, let $X_{v},X_{p},X_{e}\in R^{T\times d}$ denote the visual, physiological, and environmental features after linear projection. For notational simplicity, we describe the update of the visual branch using cross-attention to the physiological and environmental branches; the other branches are handled analogously. We define linear projections

(16) $$Q_{v}=X_{v}W_{q},\;\;\;K_{p}=X_{p}W_{k},\;\;\;V_{p}=X_{p}W_{v},\;\;\;K_{e}=X_{e}W_{k},\;\;\;V_{e}=X_{e}W_{v},$$

where $W_{q},W_{k},W_{v}\in R^{d\times d_{h}}$ are learnable projection matrices shared across modalities within a block. Cross-modal attention from modality *p* to modality *v* is computed as

(17) $$A_{v\leftarrow p}=\mathrm{Softmax}\;\left(\frac{Q_{v}K_{p}^{⊤}}{\sqrt{d_{h}}}\right)V_{p},$$

and similarly for attention from modality *e* to modality *v*,

(18) $$A_{v\leftarrow e}=\mathrm{Softmax}\;\left(\frac{Q_{v}K_{e}^{⊤}}{\sqrt{d_{h}}}\right)V_{e}.$$

The Softmax(·) operator is applied row-wise over the temporal dimension to yield attention weights for each time step. The updated visual representation is given by

(19) $$Y_{v}=X_{v}+\alpha_{p}A_{v\leftarrow p}+\alpha_{e}A_{v\leftarrow e},$$

where $\alpha_{p}$ and $\alpha_{e}$ are learnable scalar coefficients satisfying $\alpha_{p}+\alpha_{e}=1$ and controlling the relative contribution of physiological and environmental information. In this way, the visual branch can adaptively integrate cues such as physiological rhythm changes and environmental disturbances at each time step. The same procedure is used to update $Y_{p}$ and $Y_{e}$ by exchanging roles of the modalities. After normalization and concatenation along the channel axis, we obtain a fused representation $F\in R^{T\times 3d}$, which is then projected back to T×d through an MLP and forwarded to the final prediction head.

From an information-theoretic perspective, CMAF is designed to increase the mutual information among modalities. Let I(X;Y) denote the mutual information between two random variables. While single-modal self-attention mainly enhances $I(X_{v};X_{v})$, $I(X_{p};X_{p})$, and $I(X_{e};X_{e})$, CMAF aims to strengthen cross-modal terms such as $I(X_{v};X_{p})$, $I(X_{v};X_{e})$, and $I(X_{p};X_{e})$. A common variational lower bound for $I(X_{i};X_{j})$ can be written as

(20) $$I\left(X_{i};X_{j}\right)\geq E_{\left(x_{i},x_{j}\right)}\left[\log D_{\theta }\left(x_{i},x_{j}\right)\right]-\log E_{x_{i}}E_{x_{j}}\left[\exp(D_{\theta }\left(x_{i},x_{j}\right))\right],$$

where $D_{\theta }\left(\cdot ,\cdot \right)$ is a learnable critic function. In our setting, the attention score matrices implicitly play a similar role to $D_{\theta }$ by assigning higher weights to modality pairs that are more informative for the task, thereby encouraging the network to align semantically consistent patterns across modalities.

When combined with the preceding SNAM module, CMAF operates in a rectification-before-alignment manner. SNAM first enhances the signal-to-noise ratio of each modality and suppresses corrupted channels, and CMAF then performs cross-modal alignment and fusion on these rectified features. Together with the edge-aware knowledge distillation mechanism, which regularizes the fused representations via a high-capacity teacher network, this hierarchical design yields a robust semantic-fusion core that can handle noisy, asynchronous, and heterogeneous multimodal signals in resource-constrained educational environments.

#### 3.4.4. Edge-Aware Knowledge Distillation

**Figure 4:**
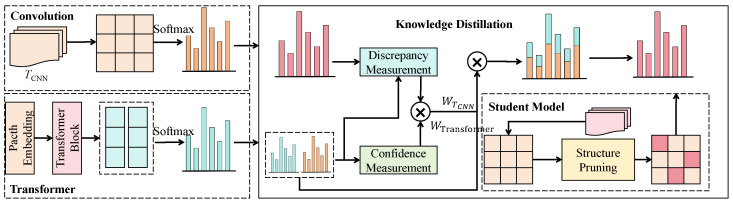
Illustration of the edge-aware knowledge distillation (EAKD) module.

The edge-aware knowledge distillation (EAKD) module aims to balance lightweight feature extraction and high-accuracy prediction on edge devices by introducing a dual-teacher distillation strategy. In this way, the lightweight student network deployed on devices such as Jetson Nano and Raspberry Pi can inherit the decision capability of more expressive teacher models trained on high-performance servers. As illustrated in Figure 4, EAKD involves two teachers and one student: a CNN teacher ($T_{\mathrm{CNN}}$), a ViT teacher, and a lightweight CNN student (denoted as $S_{\mathrm{CNN}}$). The teacher models possess stronger local and global modeling capacity, whereas the student model contains only essential convolutional and attention layers and is designed to meet edge-computing constraints.

To adaptively combine the two teachers, we adopt a discrepancy-aware teacher weighting (DATW) scheme. For each teacher k∈{CNN,ViT}, we define a confidence score

(21) $$C_{k}\left(x\right)=\max_{c}\;p_{T_{k},c}\left(x\right),$$

that reflects how certain the teacher is about its prediction, and a discrepancy measure

(22) $$D_{k}\left(x\right)=∥z_{T_{k}}\left(x\right)-z_{S}\left(x\right){∥}_{2},$$

that reflects how far the student currently deviates from that teacher in logit space. The unnormalized teacher weight is then given by

(23) $${\tilde{\omega }}_{T_{k}}\left(x\right)=\frac{C_{k}\left(x\right)}{D_{k}\left(x\right)+\epsilon },$$

where ϵ>0 is a small constant to avoid division by zero. The normalized weights are obtained as

(24) $$\omega_{T_{k}}\left(x\right)=\frac{{\tilde{\omega }}_{T_{k}}\left(x\right)}{{\tilde{\omega }}_{T_{\mathrm{CNN}}}\left(x\right)+{\tilde{\omega }}_{T_{\mathrm{ViT}}}\left(x\right)},$$

so that $\omega_{T_{\mathrm{CNN}}}\left(x\right)+\omega_{T_{\mathrm{ViT}}}\left(x\right)=1$ for all *x*. The combined teacher target, illustrated as $Z_{target}\left(x\right)$ in Figure 4, is a convex combination of the two teacher logits:

(25) $$z_{\mathrm{target}}\left(x\right)=\omega_{T_{\mathrm{CNN}}}\left(x\right)\;z_{T_{\mathrm{CNN}}}\left(x\right)+\omega_{T_{\mathrm{ViT}}}\left(x\right)\;z_{T_{\mathrm{ViT}}}\left(x\right).$$

The EAKD loss integrates logit matching and distribution-level distillation. The logit-level term encourages the student to approximate the combined teacher target $Z_{target}\left(x\right)$,

(26) $$L_{\mathrm{logit}}=∥z_{S}\left(x\right)-z_{\mathrm{target}}\left(x\right){∥}_{2}^{2},$$

and the distribution-level term uses Kullback–Leibler divergence to pull the student probabilities toward the teacher probabilities,

(27) $$L_{\mathrm{KL}}=\sum_{k\in \{\mathrm{CNN},\mathrm{ViT}\}}\mathrm{KL}\left(p_{T_{k}}\left(x\right)∥p_{S}\left(x\right)\right).$$

The overall EAKD objective is then written as

(28) $$L_{\mathrm{EAKD}}=\eta \;L_{\mathrm{logit}}+\left(1-\eta \right)\;L_{\mathrm{KL}},$$

where η∈[0,1] is a balancing coefficient tuned on the validation set. When the student already matches a given teacher well (small $D_{k}\left(x\right)$), the corresponding weight $\omega_{T_{k}}\left(x\right)$ increases, and that teacher dominates the distillation signal; when the discrepancy is large or the teacher is uncertain (low $C_{k}\left(x\right)$), its influence is automatically reduced.

By combining structurally different teachers (CNN and ViT), explicitly modeling teacher confidence and student–teacher discrepancy, and constraining the student to a lightweight architecture, EAKD enables LightEdu-Net to approach the decision quality of high-capacity models while maintaining low computational cost. In the intelligent tutoring task considered here, this module increases recognition accuracy on low-power platforms, reduces inference latency, and alleviates dependence on high-bandwidth transmission, thereby achieving a favorable balance between computation and intelligence for edge-deployed educational models.

## 4. Results and Discussion

### 4.1. Experimental Settings

#### 4.1.1. Platforms and Parameters

The experimental hardware platforms employed in this study encompass three typical categories of edge and near-edge computing devices to comprehensively validate the model’s adaptability and performance under varying computational capacities. NVIDIA Jetson Nano, equipped with a 128-core Maxwell GPU, serves as a representative low-power embedded platform commonly available in rural schools. Raspberry Pi 4B contains a quad-core ARM Cortex-A72 CPU and performs inference purely on processor resources, aligning with extremely low-compute deployment scenarios. Intel NUC, as a lightweight desktop-class system, integrates a multi-core CPU with higher clock frequency, enabling evaluation of real-time performance and energy consumption under moderate computational conditions. Deployment across this hardware spectrum provides a realistic demonstration of the computational heterogeneity and operational demands likely encountered in real-world educational environments.

Regarding software configuration, the experiments are conducted using PyTorch 2.2 as the primary deep learning framework for model construction and training. GPU-accelerated inference is enabled through CUDA 12.1 to ensure efficient computation for convolutional and Transformer-based operations. Python 3.10 is adopted as the primary programming language to support data preprocessing, model training, visualization, and general experimental workflow management. All components are executed within an Ubuntu environment to ensure dependency compatibility and stable model deployment, thereby facilitating consistent migration to edge devices. For hyperparameter configuration, the constructed dataset is partitioned into training, validation, and test sets with a ratio of 7:2:1, ensuring adequate multimodal feature learning during training while preserving sufficient samples for performance assessment. A mini-batch strategy with batch size = 32 is adopted to balance memory consumption and stable gradient updates. The learning rate is set to $1\times 10^{-4}$ and optimized with the Adam optimizer to achieve adaptive gradient updates while maintaining stable convergence. A total of 100 training epochs is used to allow sufficient convergence time. To further evaluate the generalization capability under multimodal input conditions, a five-fold cross-validation strategy is incorporated. Specifically, the dataset is randomly divided into five subsets, with four subsets used for training and one for validation in each iteration. After five iterations, the averaged performance metrics are obtained, reducing bias introduced by data partitioning and yielding a more reliable estimation of model performance.

#### 4.1.2. Baseline Models and Evaluation Metrics

A set of representative lightweight and multimodal baseline models is selected for comparison, including MobileNetV3 + SVM [45,46], ResNet [47], EfficientNet-lite [48], Tiny-Transformer [49], and CLIP-Fusion [50]. MobileNetV3 + SVM combines an efficient feature extraction backbone with a traditional classifier, offering advantages in structural simplicity and training stability. ResNet, as a classical residual architecture, provides strong robustness and consistent performance. EfficientNet-lite adopts a compound scaling strategy across depth, width, and resolution, achieving high parameter efficiency with relatively strong accuracy. Tiny-Transformer employs a lightweight self-attention structure by reducing attention heads and channel dimensions, enabling long-range dependency modeling under constrained computational budgets. CLIP-Fusion exploits pretrained vision–language alignment models as multimodal encoders, enabling feature concatenation or matching across modalities and providing strong cross-modal transferability. For all baselines, network depth, width, learning rate, and batch size are selected according to their original papers or official lightweight configurations and then fine-tuned on a held-out validation set to ensure a fair comparison under similar computational budgets.

The evaluation in this study covers classification metrics (accuracy, precision, recall, F1-score), efficiency metrics (model size, FLOPs, inference latency, power consumption), and robustness metrics (performance degradation under noise, expressed as ΔAccuracy), providing a comprehensive assessment of model suitability in resource-constrained educational environments. The mathematical definitions of the evaluation metrics are as follows:

(29) $$Accuracy=\frac{TP+TN}{TP+TN+FP+FN},$$

(30) $$Precision=\frac{TP}{TP+FP},$$

(31) $$Recall=\frac{TP}{TP+FN},$$

(32) $$F1=2\times \frac{Precision\times Recall}{Precision+Recall},$$

(33) $$\Delta Accuracy=Accuracy_{clean}-Accuracy_{noise}.$$

The symbols in these equations are defined as follows: TP denotes the number of true positives, representing instances correctly identified as positive; TN denotes true negatives, referring to instances correctly identified as negative; FP denotes false positives, referring to negative instances incorrectly classified as positive; FN denotes false negatives, representing positive instances incorrectly classified as negative. $Accuracy_{clean}$ denotes the accuracy obtained under noise-free conditions, whereas $Accuracy_{noise}$ represents the accuracy under specific noise perturbations, thereby reflecting the model’s sensitivity to noise.

### 4.2. Comparison of LightEdu-Net and Baseline Models

The objective of this experiment is to verify whether the proposed LightEdu-Net can maintain high recognition accuracy while remaining lightweight and computationally efficient in multimodal student-state recognition tasks, thereby satisfying real-time inference demands on resource-constrained edge devices. By comparing LightEdu-Net with five representative lightweight and multimodal baseline models, a comprehensive evaluation of the trade-offs among classification capacity, representational ability, and computational efficiency under different architectural designs can be achieved.

**Figure 5:**
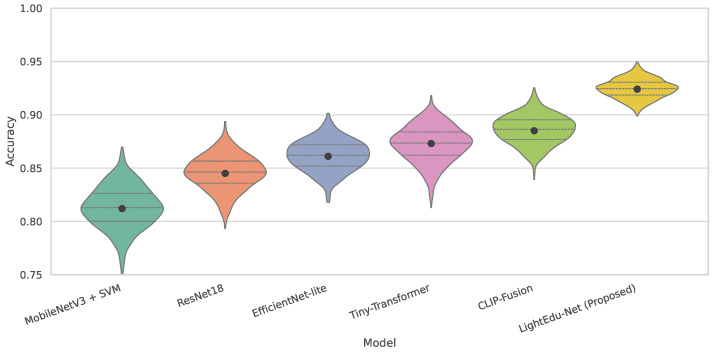
The violin plot of accuracy comparision results across different models.

As shown in Table 2 and Figure 5, conventional convolution-based models such as MobileNetV3 + SVM and ResNet18 exhibit considerably lower accuracy and recall. Their feature extraction capacity is restricted by shallow convolutional kernels with limited receptive fields, which are insufficient for capturing long-range temporal dependencies and subtle cross-modal cues, such as gradual attention changes or cumulative physiological fatigue. EfficientNet-lite and Tiny-Transformer provide stronger representational capacity but still fall short in explicitly modeling and exploiting complementary information across visual, physiological, and environmental inputs. CLIP-Fusion, as a large pretrained multimodal model, achieves relatively high accuracy; however, its large parameter scale and high FLOPs make deployment on edge devices impractical in rural classroom settings. In contrast, LightEdu-Net achieves the best performance across all metrics, with particularly notable gains in F1-score and recall, because SNAM improves the signal-to-noise ratio at the feature level, CMAF explicitly aligns and fuses modalities through cross-attention, and EAKD transfers knowledge from high-capacity teachers into a compact student. This combination yields a model that is both more discriminative for complex multimodal patterns and better suited to low-power edge deployment.

From a theoretical perspective, performance differences arise from variations in intrinsic structural design and mathematical representational capacity. MobileNetV3 + SVM and ResNet18 rely on local convolutional kernels and lack mechanisms for long-range or multimodal dependency modeling, resulting in representational bottlenecks when handling high-dimensional heterogeneous features from visual, physiological, and environmental signals. EfficientNet-lite increases network capacity through compound scaling, yet it primarily captures patterns within the visual domain, limiting cross-modal fusion ability. Tiny-Transformer can model long-range dependencies but constrains attention computation within the visual modality, preventing deeper multimodal interaction. CLIP-Fusion optimizes a cross-modal embedding space through large-scale pretraining but suffers from high-dimensional embeddings and dense attention structures, resulting in prohibitive computational cost for edge inference. LightEdu-Net achieves superior performance by incorporating a sensor noise adaptive module (SNAM) to enhance feature stability, using a cross-modal attention fusion (CMAF) module to maximize mutual information across modalities, and adopting an edge-aware knowledge distillation (EAKD) mechanism to compress the teacher model’s decision boundaries into a lightweight student network. This combination allows the lightweight model to approximate the expressiveness of a high-capacity model while maintaining efficiency, thereby achieving optimal performance under resource constraints.

### 4.3. Ablation Study

This experiment evaluates whether the overall performance of LightEdu-Net relies on its core modules and quantifies the contributions of each component to multimodal recognition capability and noise robustness.

As shown in Table 3, the three unimodal variants (Visual-only, Physiological-only, and Environmental-only) all perform noticeably worse than the full multimodal LightEdu-Net, indicating that each single modality provides only partial information and that robust student-state recognition benefits from integrating complementary cues. Visual-only typically captures posture and facial dynamics but lacks direct information about internal arousal and external disturbances; Physiological-only is sensitive to fatigue and stress but cannot distinguish specific classroom behaviors; Environmental-only reflects illumination and noise changes but is insufficient for fine-grained engagement recognition. The performance of these variants highlights the necessity of multimodal fusion. retaining only the backbone network leads to a significant decline across all performance metrics, demonstrating that shallow convolutional or limited long-range modeling is insufficient for capturing deep multimodal dependencies. Removing SNAM results in the most severe performance degradation, especially under noise level 0.3, indicating that illumination variations, sensor jitter, and physiological noise—frequently present in real-world rural classrooms—substantially affect feature stability. Removing CMAF moderately reduces performance, confirming that cross-modal attention plays a critical role in exploiting complementary information among modalities. Removing EAKD leads to a mild performance drop, showing that the distillation mechanism effectively enhances the expressive capacity of the lightweight student model without increasing computational cost. From a theoretical standpoint, SNAM stabilizes temporal and spectral characteristics by reducing distributional shifts from noise perturbations. CMAF maximizes mutual information across modalities, equivalent to learning conditional dependency structures in a high-dimensional feature space. Removing CMAF degrades joint representations into weakly coupled features. EAKD provides a richer teacher-defined feature space, allowing the lightweight model to maintain high-level decision boundaries; removing it reduces classification granularity and generalization capability.

### 4.4. Deployment Performance

This experiment evaluates the deployability and stability of LightEdu-Net under different computational resources by testing latency, throughput, power consumption, and accuracy on three representative edge and near-edge platforms. The goal is to determine whether the model satisfies real-time, energy-efficient, and reliable recognition requirements in practical educational environments.

As shown in Table 4, Jetson Nano, despite limited computational capability, achieves relatively high throughput and low latency due to the lightweight convolutional architecture and compact fused features, while maintaining server-level accuracy. Raspberry Pi 4B, relying solely on CPU inference, exhibits higher latency but retains stable accuracy under low power consumption, confirming that the model design minimizes convolutional depth and attention complexity to accommodate CPU-based sequential operations. Intel NUC, with higher clock frequencies and multi-core processing, provides the lowest latency and highest throughput, indicating strong scalability on more capable platforms.

### 4.5. Discussion

#### 4.5.1. Effectiveness of LightEdu-Net on Core Challenges

The experimental results validate that LightEdu-Net effectively addresses the key challenges of noisy sensing, multimodal fusion, and edge deployability in rural classroom environments. As shown in Table 2 and Figure 5, LightEdu-Net achieves the highest accuracy, precision, recall, and F1-score among all compared methods while maintaining a moderate model size and FLOPs, indicating a favorable balance between representational capacity and computational efficiency. The ablation results in Table 3 further clarify the contribution of each core component: the “Backbone Only” variant exhibits pronounced performance degradation, removing SNAM causes the largest drop and severe sensitivity to noise, removing CMAF weakens the exploitation of complementary multimodal information, and removing EAKD reduces performance despite unchanged inference cost. Together with the deployment results in Table 4, which show that LightEdu-Net meets latency, throughput, and power constraints on Jetson Nano, Raspberry Pi 4B, and Intel NUC, these findings confirm that SNAM, CMAF, and EAKD work synergistically to provide robust, accurate, and deployable multimodal perception under realistic edge-computing conditions.

From an educational perspective, field feedback from teachers suggests that the system’s continuous indicators of attention, fatigue, and participation provide actionable information that is difficult to obtain in large classes, enabling timely adjustments in pacing, interaction strategies, and classroom management. This indicates that LightEdu-Net can help increase the effective use of limited teaching resources and improve instructional efficiency in rural and under-resourced schools. At the same time, the sensitivity of multimodal behavioral and physiological data highlights the need for privacy-preserving extensions, such as federated learning, in which model updates rather than raw data are shared across schools. Future work will also involve more fine-grained power-consumption measurements under varying workloads (e.g., different class sizes and sampling rates) to better assess long-term sustainability. Overall, the current experiments show that the core challenges identified in this study—sensor noise, multimodal fusion, and edge deployment—are substantially mitigated, and that LightEdu-Net provides a practical and scalable foundation for multimodal perception in real classroom environments.

#### 4.5.2. Classroom Usage, Annotation Protocol, and Educational Impact

Beyond quantitative performance, it is important to clarify how LightEdu-Net operates at the behavioral level and how its outputs are obtained. In our implementation, the model predicts discrete engagement-related states, including multi-level attention labels (high/medium/low), fatigue levels (alert/mild/high), and participation indicators (active/passive), derived from the fused multimodal representation produced by CMAF and generated at regular time intervals for each student and the class as a whole. Ground truth labels are constructed through an offline annotation protocol: synchronized video, physiological signals, and classroom logs are reviewed by trained annotators with educational backgrounds, who assign attention, fatigue, and participation levels based on observable cues such as gaze orientation, posture, facial expressions, interaction frequency, and characteristic changes in physiological signals. Each segment is labeled independently by at least two annotators, and disagreements are resolved by consensus, ensuring that the supervisory signals correspond to pedagogically meaningful states rather than purely low-level patterns.

In real deployments, the outputs of LightEdu-Net are delivered to teachers via a lightweight classroom dashboard designed to impose minimal cognitive load. At the individual level, the interface presents smoothed timelines of attention and fatigue using simple color-coded bars without exposing raw physiological values, while at the class level it provides aggregated indicators (e.g., proportion of students with low attention, average fatigue level) as trend curves or compact heatmaps. This allows teachers to react during class to sudden drops in engagement by adjusting pacing, questioning strategies, or instructional modalities, and to reflect after class on when and where engagement declined to inform lesson redesign and targeted support. Over longer periods, aggregated statistics across lessons help administrators identify structural inefficiencies (such as overloaded time slots or problematic subject scheduling) and optimize timetables and resource allocation. From an educational economics perspective, this workflow converts previously unobservable behavioral processes into measurable process data, enabling more efficient use of limited teaching resources and supporting equity-oriented decision-making in resource-constrained schools, with modest hardware cost due to edge-based deployment.

### 4.6. Limitations and Future Work

Although the proposed study provides a comprehensive evaluation of multimodal student-state recognition and edge deployment, several limitations remain with respect to the core challenges of noisy sensing, multimodal fusion, and resource-constrained inference, and these should be addressed in future research. First, the dataset was collected from multiple rural primary schools and one county-level middle school and primarily reflects typical classroom behaviors in this specific regional and cultural context. While the experiments demonstrate that LightEdu-Net is robust to sensor noise and heterogeneous modalities under these conditions, its generalization ability has not yet been fully validated for other subject types (e.g., laboratory sessions, project-based learning), special learning behaviors (e.g., students with learning difficulties), or classrooms with different cultural, linguistic, or curriculum backgrounds. In addition, the current dataset, though multimodal, still focuses on a limited set of physiological and environmental variables. Future work should expand data collection across more diverse schools and regions, include richer behavioral and contextual modalities, and explore unified annotation and preprocessing standards so that the noise-resilient and fusion mechanisms of LightEdu-Net can be systematically evaluated and adapted in broader settings. Second, although the proposed architecture achieves real-time inference on mainstream edge platforms such as Jetson Nano, Raspberry Pi 4B, and Intel NUC, the current design is primarily optimized for these mid-range devices. Under ultra-low-power or highly constrained hardware (e.g., wearable devices, low-end tablets, or legacy educational terminals), the computational cost of cross-modal attention and feature alignment remains a bottleneck, and the latency may no longer satisfy strict real-time requirements. The present SNAM, CMAF, and EAKD modules effectively alleviate the original deployment challenge within the tested resource envelope, but they do not yet provide a principled solution for the extreme edge regime. Future research should therefore investigate more efficient low-rank and sparse attention structures, event-driven or adaptive-inference strategies that selectively update only salient segments, and lighter distillation frameworks tailored to on-device training or continual learning. These directions will be important for further reducing computational and energy overhead while preserving the robustness and multimodal discriminative capability demonstrated in this work.

## 5. Conclusions

This study addresses a core challenge in deploying intelligent tutoring systems within resource-constrained educational environments, namely, how to perform reliable multimodal student-state recognition under sensor noise, heterogeneous inputs, and limited computational resources. To tackle this problem, we propose LightEdu-Net, a lightweight multimodal framework that combines a sensor-noise adaptive module to stabilize feature quality, a cross-modal attention fusion mechanism to enhance information interaction across visual, physiological, and environmental signals, and an edge-aware knowledge distillation strategy to preserve discriminative capacity on low-power devices. Experimental evaluations demonstrate clear progress in overcoming the identified challenges: LightEdu-Net achieves 92.4% accuracy with balanced precision and recall and maintains stable performance under noise perturbation while supporting real-time inference on Jetson Nano, Raspberry Pi 4B, and Intel NUC. These results confirm that the proposed solutions improve robustness, fusion expressiveness, and deployability, providing a viable pathway for multimodal perception in rural and under-resourced classrooms.

Despite these advances, several limitations remain when addressing the deployment challenge in its full scope. The current approach has been validated primarily on a limited set of schools and typical classroom settings, and its generalization to diverse learning activities, cultural contexts, or extreme hardware constraints has not been fully established. Moreover, although latency and power consumption are acceptable for mainstream edge devices, further reductions will be required for ultra-low-power platforms such as wearable systems or legacy terminals. Future work will therefore focus on expanding multimodal datasets across broader regions, investigating more efficient attention and distillation mechanisms, and exploring privacy-preserving training strategies such as federated learning to enable scalable deployment across multiple schools. These directions will further enhance the practicality and sustainability of LightEdu-Net and accelerate its application in real-world educational environments.

## Figures and Tables

**Table 1:** Statistics of the multimodal educational behavior dataset.

Data Type	Source	Sampling Rate/FPS	Sample Count
Visual Data	Classroom fixed cameras	30 FPS	2,160,000 frames
Physiological Data	Wearable sensors (HR, EDA, TEMP, SpO_2_)	10 Hz	360,000 sets
Environmental Data	LUX/noise/temperature/humidity sensors	1 Hz	540,000 entries

**Table 2:** Comparison of LightEdu-Net and baseline models in classification performance and efficiency.

Model	Accuracy	Precision	Recall	F1-Score	Model Size (MB)	FLOPs (G)
MobileNetV3 + SVM	81.2 ± 0.6%	78.9 ± 0.5%	77.5 ± 0.7%	78.2 ± 0.6%	9.8	0.65
ResNet18	84.5 ± 0.4%	83.2 ± 0.6%	82.7 ± 0.5%	82.9 ± 0.4%	44.7	1.82
EfficientNet-lite	86.1 ± 0.5%	85.4 ± 0.4%	84.2 ± 0.6%	84.7 ± 0.5%	13.5	0.92
Tiny-Transformer	87.3 ± 0.4%	86.1 ± 0.5%	85.9 ± 0.3%	86.0 ± 0.4%	15.8	1.35
CLIP-Fusion	88.5 ± 0.3%	87.4 ± 0.4%	87.1 ± 0.3%	87.2 ± 0.3%	328.3	4.20
LightEdu-Net (Proposed)	92.4 ± 0.2%	91.7 ± 0.3%	91.2 ± 0.2%	91.4 ± 0.3%	34.5	1.05

**Table 3:** Ablation study of LightEdu-Net showing the contribution of each module and input configuration.

Model Variant	Accuracy	Precision	Recall	F1-Score	ΔAccuracy (Noise = 0.3)
Visual-only	87.3 ± 0.5%	86.5 ± 0.4%	86.1 ± 0.6%	86.3 ± 0.5%	−8.2%
Physiological-only	84.6 ± 0.6%	83.8 ± 0.5%	83.1 ± 0.7%	83.4 ± 0.6%	−10.1%
Environmental-only	82.9 ± 0.7%	82.1 ± 0.6%	81.4 ± 0.8%	81.7 ± 0.7%	−11.6%
Without SNAM	88.1 ± 0.6%	87.2 ± 0.5%	86.7 ± 0.7%	86.9 ± 0.6%	−7.8%
Without CMAF	89.4 ± 0.5%	88.1 ± 0.4%	87.9 ± 0.6%	88.0 ± 0.5%	−5.2%
Without EAKD	90.2 ± 0.4%	89.5 ± 0.5%	89.1 ± 0.3%	89.2 ± 0.4%	−2.1%
Backbone Only	85.7 ± 0.7%	84.9 ± 0.6%	84.1 ± 0.8%	84.5 ± 0.7%	−10.4%
Full LightEdu-Net	92.4 ± 0.3%	91.7 ± 0.3%	91.2 ± 0.2%	91.4 ± 0.3%	−1.1%

**Table 4:** Deployment performance of LightEdu-Net across different edge and near-edge platforms.

Hardware Platform	Mean Latency (ms)	Throughput (FPS)	Power Consumption (W)	Accuracy
Jetson Nano	42.8	23.4	7.1	92.4%
Raspberry Pi 4B	108.5	9.1	5.3	92.1%
Intel NUC	18.9	52.7	14.5	92.4%

## Data Availability

The data presented in this study are available on request from the corresponding author.

## References

[1] Kayumova M., Akbarova S., Azizova M., Hojiyeva I., Karimova F., Makhmudova F. Data Driven Teaching and Real Time Decision Making in Education Management. Proceedings of the 8th International Conference on Future Networks & Distributed Systems.

[2] Trabelsi Z., Alnajjar F., Parambil M.M.A., Gochoo M., Ali L. (2023). Real-time attention monitoring system for classroom: A deep learning approach for student’s behavior recognition. Big Data Cogn. Comput..

[3] Wei N., Yang F., Muthu B., Shanthini A. (2022). Human machine interaction-assisted smart educational system for rural children. Comput. Electr. Eng..

[4] TS A., Guddeti R.M.R. (2020). Automatic detection of students’ affective states in classroom environment using hybrid convolutional neural networks. Educ. Inf. Technol..

[5] Nye B.D. (2015). Intelligent tutoring systems by and for the developing world: A review of trends and approaches for educational technology in a global context. Int. J. Artif. Intell. Educ..

[6] Hwang K.A., Yang C.H. (2009). Automated inattention and fatigue detection system in distance education for elementary school students. J. Educ. Technol. Soc..

[7] Pabba C., Kumar P. (2022). An intelligent system for monitoring students’ engagement in large classroom teaching through facial expression recognition. Expert Syst..

[8] Alsayigh H.K.S., Khidhir A.S.M. (2024). Using IoT to predict student attention levels in e-learning classes: A review. AIP Conf. Proc..

[9] Gupta S., Kumar P., Tekchandani R. (2023). A machine learning-based decision support system for temporal human cognitive state estimation during online education using wearable physiological monitoring devices. Decis. Anal. J..

[10] Chen S., Hu Z., Li S., Hu X., Liu G., Wen W. (2021). Autonomic nervous pattern recognition of students’ learning states in real classroom situation. IEEE Trans. Comput. Soc. Syst..

[11] Miao Y. (2021). Online and offline mixed intelligent teaching assistant mode of english based on mobile information system. Mob. Inf. Syst..

[12] Sajja R., Sermet Y., Cikmaz M., Cwiertny D., Demir I. (2024). Artificial intelligence-enabled intelligent assistant for personalized and adaptive learning in higher education. Information.

[13] Liu Y., Chen L., Yao Z. (2022). The application of artificial intelligence assistant to deep learning in teachers’ teaching and students’ learning processes. Front. Psychol..

[14] Yang Y. (2025). Design and Implementation of Algorithm-Based Intelligent Auxiliary System for Physical Education Teaching Equipment. Int. J. High Speed Electron. Syst..

[15] Wang J. (2024). Generative Pre-Trained Transformer-4 based on English Assistant Teaching System in Higher Vocational Colleges. Proceedings of the 2024 First International Conference on Software, Systems and Information Technology (SSITCON).

[16] Wang J., Lu T., Li L., Huang D. (2024). Enhancing personalized search with AI: A hybrid approach integrating deep learning and cloud computing. J. Adv. Comput. Syst..

[17] Ji X., Sun L., Huang K. (2025). The construction and implementation direction of personalized learning model based on multimodal data fusion in the context of intelligent education. Cogn. Syst. Res..

[18] Zhang Y., He S., Wa S., Zong Z., Lin J., Fan D., Fu J., Lv C. (2022). Symmetry GAN detection network: An automatic one-stage high-accuracy detection network for various types of lesions on CT images. Symmetry.

[19] Rashid H.A., Ovi P.R., Busart C., Gangopadhyay A., Mohsenin T. (2022). Tinym2net: A flexible system algorithm co-designed multimodal learning framework for tiny devices. arXiv.

[20] Jiang D., Shen Z., Zheng Q., Zhang T., Xiang W., Jin J. (2025). Farm-LightSeek: An Edge-centric Multimodal Agricultural IoT Data Analytics Framework with Lightweight LLMs. IEEE Internet Things Mag..

[21] Xiong Y., Guo X., Xu J. (2024). CNN-Transformer: A deep learning method for automatically identifying learning engagement. Educ. Inf. Technol..

[22] Cruz Y.J., Villalonga A., Castaño F., Rivas M., Haber R.E. (2024). Automated machine learning methodology for optimizing production processes in small and medium-sized enterprises. Oper. Res. Perspect..

[23] Pei Y., Lu G. (2023). Design of an intelligent educational evaluation system using deep learning. IEEE Access.

[24] Bouafoud C., Zine-Dine K., Madani A. (2024). The Evolution of Transformers in Education: A Literature Review. Proceedings of the 2024 International Conference on Circuit, Systems and Communication (ICCSC).

[25] Sun P., Gu L. (2021). Fuzzy knowledge graph system for artificial intelligence-based smart education. J. Intell. Fuzzy Syst..

[26] Dan Y., Lei Z., Gu Y., Li Y., Yin J., Lin J., Ye L., Tie Z., Zhou Y., Wang Y. (2023). Educhat: A large-scale language model-based chatbot system for intelligent education. arXiv.

[27] Xu Q., Wu Y., Zheng H., Yan H., Wu H., Qian Y., Wu Y., Liu B. (2025). Standardization in artificial general intelligence model for education. Comput. Stand. Interfaces.

[28] Gan W., Dao M.S., Zettsu K., Sun Y. IoT-based multimodal analysis for smart education: Current status, challenges and opportunities. Proceedings of the 3rd ACM Workshop on Intelligent Cross-Data Analysis and Retrieval.

[29] Li C., Liu C., Ju W., Zhong Y., Li Y. (2025). Prediction of teaching quality in the context of smart education: Application of multimodal data fusion and complex network topology structure. Discov. Artif. Intell..

[30] Chango W., Lara J.A., Cerezo R., Romero C. (2022). A review on data fusion in multimodal learning analytics and educational data mining. Wiley Interdiscip. Rev. Data Min. Knowl. Discov..

[31] Luo Z., Zheng C., Gong J., Chen S., Luo Y., Yi Y. (2023). 3DLIM: Intelligent analysis of students’ learning interest by using multimodal fusion technology. Educ. Inf. Technol..

[32] Shi W., Nie Z., Shi Y. (2024). Application and Challenges of Multi-Modal Data Fusion in Adaptive Learning System. Proceedings of the 2024 6th International Conference on Computer Science and Technologies in Education (CSTE).

[33] Qianyi Z., Zhiqiang L. (2023). Research on multimodal based learning evaluation method in smart classroom. Learn. Motiv..

[34] Zhao F., Zhang C., Geng B. (2024). Deep multimodal data fusion. ACM Comput. Surv..

[35] Dai Z., Zhang Q., Zhao L., Zhu X., Zhou D. (2023). Cloud-edge computing technology-based internet of things system for smart classroom environment. Int. J. Emerg. Technol. Learn. (iJET).

[36] Kumari K.A., Sadasivam G.S., Dharani D., Niranjanamurthy M. (2021). Edge Computing: Fundamentals, Advances and Applications.

[37] Shuvo M.M.H., Islam S.K., Cheng J., Morshed B.I. (2022). Efficient acceleration of deep learning inference on resource-constrained edge devices: A review. Proc. IEEE.

[38] Bauravindah A., Fudholi D.H. (2024). Lightweight models for real-time steganalysis: A Comparison of MobileNet, ShuffleNet, and EfficientNet. J. RESTI (Rekayasa Sist. Dan Teknol. Inf.).

[39] Ahmadi M., Mirmahboub B., Karimi N., Khadivi P., Samavi S. (2025). AI-Assisted Breast Cancer Classification: A Deep Learning Model Integrating MobileNet and ShuffleNet Features. Proceedings of the 2025 IEEE World AI IoT Congress (AIIoT).

[40] Khediri N., Ben Ammar M., Kherallah M. (2024). A real-time multimodal intelligent tutoring emotion recognition system (MITERS). Multimed. Tools Appl..

[41] Xia C. (2025). Research on the Application of Large Language Models in Intelligent Tutoring System. Proceedings of the 2025 3rd International Conference on Image, Algorithms, and Artificial Intelligence (ICIAAI 2025).

[42] You C., Lu H., Li P., Zhao X., Yao Y. (2025). AI-Driven Intelligent Learning Companions: A Multimodal Fusion Framework for Personalized Education. Proceedings of the 2025 IEEE 34th Wireless and Optical Communications Conference (WOCC).

[43] El Hajji M., Ait Baha T., Berka A., Ait Nacer H., El Aouifi H., Es-Saady Y. (2025). An Architecture for Intelligent Tutoring in Virtual Reality: Integrating LLMs and Multimodal Interaction for Immersive Learning. Information.

[44] Jia J., He Y., Le H. (2020). A multimodal human-computer interaction system and its application in smart learning environments. Proceedings of the International Conference on Blended Learning.

[45] Cortes C., Vapnik V. (1995). Support-vector networks. Mach. Learn..

[46] Howard A., Sandler M., Chu G., Chen L.C., Chen B., Tan M., Wang W., Zhu Y., Pang R., Vasudevan V. Searching for mobilenetv3. Proceedings of the IEEE/CVF International Conference on Computer Vision.

[47] He K., Zhang X., Ren S., Sun J. Deep residual learning for image recognition. Proceedings of the IEEE Conference on Computer Vision and Pattern Recognition.

[48] Ab Wahab M.N., Nazir A., Ren A.T.Z., Noor M.H.M., Akbar M.F., Mohamed A.S.A. (2021). Efficientnet-lite and hybrid CNN-KNN implementation for facial expression recognition on raspberry Pi. IEEE Access.

[49] Barbieri L., Brambilla M., Stefanutti M., Romano C., De Carlo N., Roveri M. (2023). A tiny transformer-based anomaly detection framework for IoT solutions. IEEE Open J. Signal Process..

[50] Çökmez G.M., Zhang Y., Schroers C., Aydin T.O. (2025). CLIP-Fusion: A Spatio-Temporal Quality Metric for Frame Interpolation. Proceedings of the 2025 IEEE/CVF Winter Conference on Applications of Computer Vision (WACV).

